# Effect of numbering of return envelopes on participation, explicit refusals, and bias: experiment and meta-analysis

**DOI:** 10.1186/1471-2288-14-6

**Published:** 2014-01-15

**Authors:** Thomas V Perneger, Stéphane Cullati, Sandrine Rudaz, Thomas Agoritsas, Ralph E Schmidt, Christophe Combescure, Delphine S Courvoisier

**Affiliations:** 1 Division of Clinical Epidemiology, University Hospitals of Geneva, Geneva, Switzerland; 2 Faculty of Medicine, University of Geneva, Geneva, Switzerland; 3 Department of Clinical Epidemiology and Biostatistics, McMaster University, Hamilton, Canada; 4 Swiss Center for Affective Sciences, University of Geneva, Geneva, Switzerland

**Keywords:** Health surveys, Survey participation, Survey numbering, Bias, Sensitive topic, Meta-analysis

## Abstract

**Background:**

Tracing mail survey responses is useful for the management of reminders but may cause concerns about anonymity among prospective participants. We examined the impact of numbering return envelopes on the participation and the results of a survey on a sensitive topic among hospital staff.

**Methods:**

In a survey about regrets associated with providing healthcare conducted among hospital-based doctors and nurses, two randomly drawn subsamples were provided numbered (N = 1100) and non-numbered (N = 500) envelopes for the return of completed questionnaires. Participation, explicit refusals, and item responses were compared. We also conducted a meta-analysis of the effect of questionnaire/envelope numbering on participation in health surveys.

**Results:**

The participation rate was lower in the “numbered” group than in the “non-numbered” group (30.3% vs. 35.0%, p = 0.073), the proportion of explicit refusals was higher in the “numbered” group (23.1% vs 17.5%, p = 0.016), and the proportion of those who never returned the questionnaire was similar (46.6% vs 47.5%, p = 0.78). The means of responses differed significantly for 12 of 105 items (11.4%), which did not differ significantly from the expected frequency of type 1 errors, i.e., 5% (permutation test, p = 0.078). The meta-analysis of 7 experimental surveys (including this one) indicated that numbering is associated with a 2.4% decrease in the survey response rate (95% confidence interval 0.3% to 4.4%).

**Conclusions:**

Numbered return envelopes may reduce the response rate and increase explicit refusals to participate in a sensitive survey. Reduced participation was confirmed by a meta-analysis of randomized health surveys. There was no strong evidence of bias.

## Background

The protection of the identity of participants is an important issue in health surveys, particularly when the topic of the survey is perceived to be sensitive or intrusive by potential respondents. The reasons are both ethical and pragmatic. The risk of breaching participants’ privacy should be minimized in all research as a matter of principle. In addition, people who fear that answers may be traced back to them may choose not to participate at all, may skip the sensitive questions, or may alter their responses, consciously or unconsciously, to make them more inconspicuous or acceptable [1]. Non-response may cause selection bias, and altered answers may cause information bias (similar to social desirability bias). For these reasons health surveys are typically anonymous. However, many surveys employ a study identifier, such as a study number, that is used to track responses and manage reminders. This identifier may raise concerns among potential participants about the traceability of the respondent.

Some (but not all) older studies suggest that respondent identification may decrease participation in surveys [2-9]. Several [3-5] were marketing studies in which respondent motivations and concerns about confidentiality may differ from those of health surveys. Studies in the health or health care domain have reported small and non-significant differences in response rates associated with respondent identifiability [6-9].

We report here on a potentially sensitive study where the numbering of return envelopes may have increased non-response and possibly caused bias. The context was a mail survey of a random sample of hospital doctors and nurses regarding their experience of regrets encountered during patient care [10]. A previous qualitative study established that this was indeed a sensitive topic for most health care professionals [11]. The survey cover letter informed the potential participants that this was a research project in which participation was voluntary, that the data would not be linked with personal identifiers, and that the number on the return envelope would only be used to track responses and avoid sending unnecessary reminders. The front page instructed the recipient to check the appropriate box and send back the questionnaire without filling it in if a) the respondent had not practiced patient care in the past 5 years, b) the respondent did not wish to participate, or c) the respondent had retired. Upon receipt of the first returned questionnaires it appeared that an unusually large proportion explicitly declined to participate. To compensate for the anticipated low sample size, and to test the hypothesis that numbering of the return envelopes contributed to this problem, we selected an additional random sample of doctors and nurses. For this additional sample the return envelopes were not numbered, so that everyone received the initial mailing and the subsequent reminders.

In this report, we compare the original sample (numbered surveys) and the additional sample (non-numbered surveys) in terms of overall response rate, the proportion of explicit refusals, and the distributions of survey variables among the respondents. We also performed a meta-analysis of randomised studies of health surveys that compared participation rates for traceable and untraceable responses.

## Methods

### Design, population and data collection

In this study two consecutive samples were drawn from the same randomly ordered sampling frame, but the two samples were contacted at different moments in time, two months apart. Thus the design deviated from a classic randomized study in that the random allocation was not concurrent. The study population included all doctors and nurses employed at University Hospitals of Geneva, a large teaching hospital in Geneva, Switzerland. Separate lists of doctors and nurses were ordered at random, and the first 550 of each list were included in the initial mailing (ranks 1 through 550 on each list, total 1100). For the second mailing, an additional 250 doctors and 250 nurses were selected from the same lists (ranks 551 through 800 on each list, total 500). Each returned envelope was classified according to the participation outcome (completed, refusal, not eligible), after which completed questionnaires were assigned a new number before data entry. This guaranteed that the anonymous questionnaire database could never be linked with the survey management database that contained personal identifiers. Non-respondents of the first sample and the whole second sample were sent reminder mailings at 3 weeks, 6 weeks, and 9 weeks. These procedures, i.e., the original study protocol as well as the extension, were approved by the Ethics committee for research on human subjects of the University Hospitals of Geneva.

### Study variables

The independent variable in this analysis was the numbering of the return envelopes, versus no numbering. The dependent variables were the survey response rate among eligible participants and the proportion of explicit refusals. To analyse bias, we examined all variables measured in the questionnaire among the respondents (105 items). The first section of the questionnaire enquired about the most strongly regretted situation in the context of providing health care in the past 5 years, the second pertained to mechanisms that can be used to cope with regrets related to patient care in general, and additional sections measured life satisfaction, work satisfaction, quality of sleep, self-esteem, depression, self-rated health, and sickness leave in the past 6 months (Table 1).

**Table 1:** Domains addressed in the questionnaire, number of items per domain, and number of significant differences between numbered and non-numbered surveys

**Domain**	**Items**	**Number of significant differences**	**Numbering associated with…**
Time since event that caused regret	1	0	
Consequences for patient	9	1	Extended hospital stay *less* frequent
Type of event	7	0	
Error committed	1	0	
Intensity and manifestations of regret	19	7	*Lower* intensity (all 7 differences)
Numerical scales measuring various aspects of regret	5	1	*Lower* sense of responsibility
Number of regretted situations	1	0	
Coping strategies	31	2	*More* acceptance
			*Greater* sense of having done one’s best
Life satisfaction	5	0	
Work satisfaction	4	0	
Sleep	9	1	*Less* frequent bad dreams
Self-esteem	1	0	
Depression	10	0	
Self-rated health	1	0	
Absence from work	1	0	

### Analysis

The two groups were compared by chi-square tests on response status and other binary variables. To identify possible bias, we compared the means of 105 survey items (all except age, sex, profession and work situation), by means of Mann–Whitney tests for ordinal variables and chi-square tests for nominal variables. To assess if the number of significant test results exceeded the number expected under the general null hypothesis, we performed a random permutation test [12]. In the permutation test, in each iteration, group membership (i.e., numbered versus non-numbered envelope) was allocated at random, the 105 comparisons were performed, and the number of significant results was recorded. This was repeated 1000 times. The resulting distribution of the number of significant tests reflected the situation under the null hypothesis. The p-value for the observed number of significant tests was computed as the proportion of permutations with the same or a more extreme number of significant tests.

Analyses were conducted using SPSS version 18, and the permutation test was implemented in R [13].

### Meta-analysis

Because the impact of envelope numbering on participation was of borderline statistical significance, we performed a meta-analysis of similar studies, in order to obtain a clearer assessment of this effect. A separate protocol is not available for this *ad hoc* project, but we applied the requirements of the PRISMA statement [14] as much as possible (Additional file 1). The objective was to compare participation rates for identifiable and non-identifiable surveys. To be eligible the study had to compare respondent identification (by a code or study number) with a completely anonymous data-collection method, address a health-related topic and have a randomized or quasi-randomized design. Two of the authors (TP and DC) used the following search in PubMed and Embase: (anonymity OR anonymous OR numbering OR numbered OR coded OR tracking OR tracing) AND (response OR participation) AND (questionnaires [MeSH]) AND (randomized OR randomised OR randomly), without any limitation on year of publication or language. In addition we examined reference lists of the identified papers and relevant reviews [2]. From each study the reviewers abstracted the numbers of eligible persons and the numbers of participants in each study arm. We obtained a pooled difference in proportions of respondents using a random-effects meta-analysis, with inverse variance weights. The between-study heterogeneity was measured by the statistic I^2^. The meta-analysis was performed using Comprehensive Meta-Analysis 2.0.

## Results

### Participation and refusal rates

Of the initial 1600 potential respondents (1100 in the initial sample and 500 in the 2^nd^ sample), 116 were considered as ineligible for the survey, either because they were not involved in patient care, had retired, or their survey package was returned by the post office due to an invalid address. Among the 1484 eligible persons (1038 in the initial sample and 446 in the 2^nd^ sample), 470 (31.7%) returned a completed questionnaire, 318 (21.4%) expressed an explicit refusal, and 696 (46.9%) never returned the questionnaire.

The participation was higher and explicit refusals were fewer when the returned envelopes were not numbered (Table 2). The absolute difference in proportions of participants was 4.7% (95% CI -0.4% to 10.0%, p = 0.073) in favor of non-numbered envelopes. The proportion of explicit refusals was lower by 5.6% (95% CI 1.1% to 9.9%, p = 0.016) for non-numbered envelopes. The proportions of potential participants who did not respond were similar in the 2 groups, within a percentage point. Among returned surveys, the proportion of explicit refusal was 10.0% (95% CI 2.5% to 17.1%, p = 0.011) higher when the envelopes were numbered. The impact of numbering envelopes on the response rate was similar in doctors and nurses (stratified analysis not shown), so their results were combined.

**Table 2:** Participation and explicit refusals in a survey of patient-care related regret, for numbered versus non-numbered return envelopes

	**Numbered envelopes**	**Non-numbered envelopes**	**P value**
Returned a completed questionnaire	30.3% (314/1038)	35.0% (156/446)	0.073
Never returned the questionnaire	46.6% (484/1038)	47.5% (212/446)	0.78
Explicit refusals among eligible persons	23.1% (240/1038)	17.5% (78/446)	0.016
Explicit refusals among returned envelopes	43.3% (240/554)	33.3% (78/234)	0.011

### Participant characteristics

Among the 470 persons who completed the questionnaire there were 323 (69.5%) women, 240 (51.7%) nurses, and 312 (67.2%) employees who worked full time or nearly full-time. The mean age was 40.5 years (SD 9.2) and mean number of years since starting patient care was 15.0 years (SD 9.9). The majority (295, 63.3%) rated their health as excellent or very good, and a similar proportion (318, 68.2%) did not miss any workdays because of health problems in the past 6 months. These characteristics were similar in the 2 study groups.

### Exploring bias

**Figure 1:**
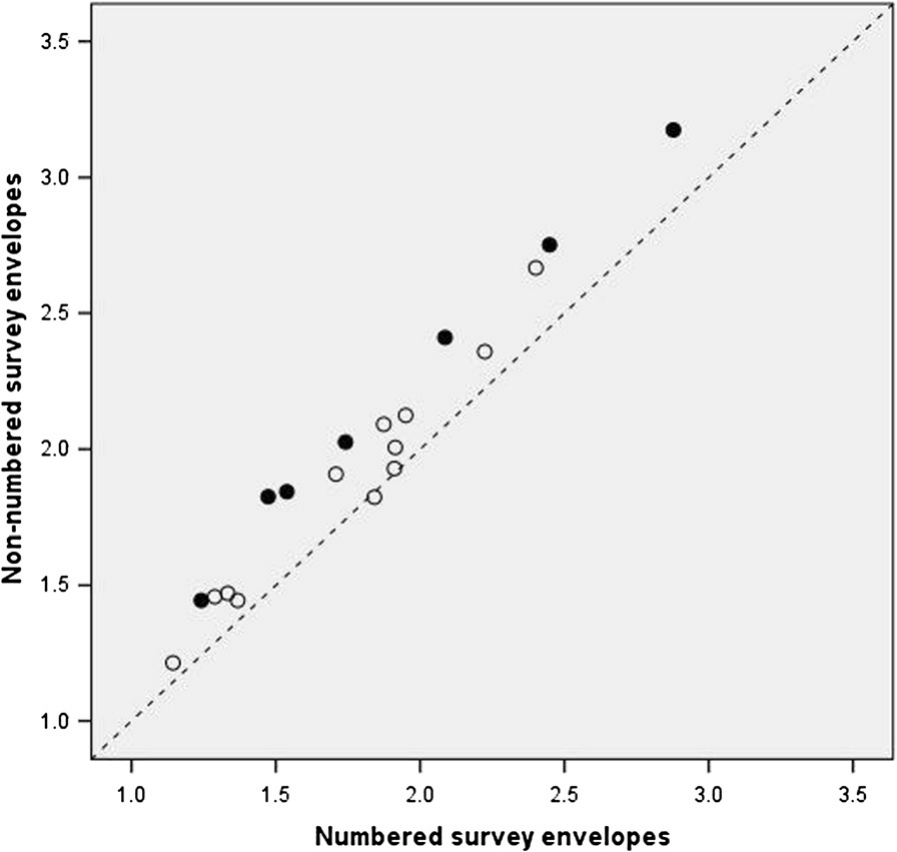
**Mean values of 19 items measuring regret intensity, in surveys with numbered versus non-numbered envelopes.** Black dots identify statistically significant differences.

The 2 groups of participants were compared on 105 survey items. Most differences were small and statistically non-significant, but 12 (11.4%) exceeded the significance level of 0.05 (Table 1). Most notably, respondents in the non-numbered group reported significantly higher intensity for 7 of 19 manifestations of their strongest regret within the past 5 years (Figure 1). This was confirmed by a higher sense of responsibility for the regretted situation (assessed on a numerical scale).

Among the 1000 random permutations of group assignment (i.e., under the null hypothesis), the number of nominally significant tests varied between 0 and 25 out of 105. In 19 permutations, the number of significant tests was 12, and in 40 others it was 13 or more (one-sided p = 0.059); in addition, in 19 permutations there were no significant tests (two-sided p = 0.078).

### Meta-analysis of participation rates

**Figure 2:**
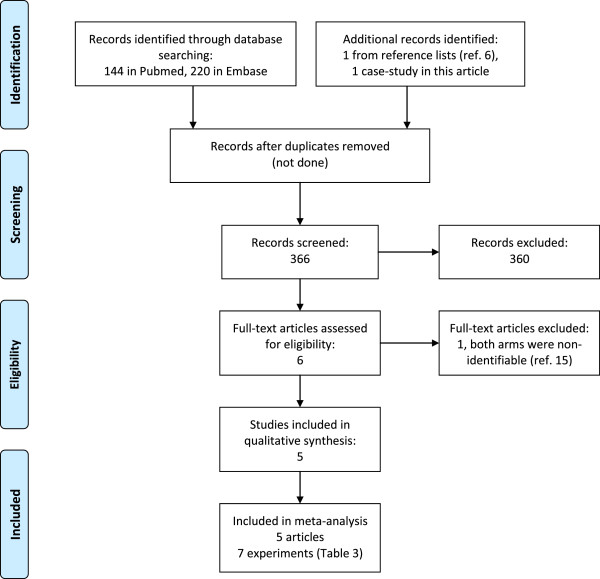
PRISMA flow diagram.

The search is depicted in Figure 2. The PubMed search identified 144 citations of which 3 were eligible [7-9], and the Embase search identified 220 papers of which two were eligible [7,9]. Both searches also initially identified a study by Asch [15], which was excluded because it compared 2 strategies that ensured anonymity: one group had no identifiers, and the other was asked to send back a coded postcard separately from the questionnaire. An additional eligible paper was retrieved from reference lists [6]. The identified articles and the abstracted results were identical for the two reviewers.

**Figure 3:**
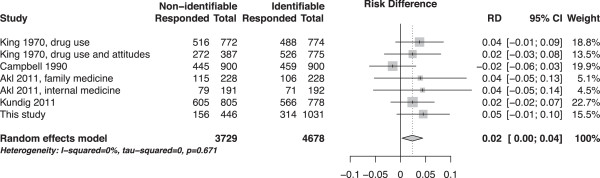
Forest plot of the difference in participation rates between identifiable and non-identifiable surveys.

The four papers [6-9] reported on 6 separate experiments. These results were combined with the results of this study (Table 3, Figure 3). The pooled difference in participation rates was 2.4% (95% confidence interval, 0.3% to 4.4%). We detected no between-study heterogeneity in the effect of numbering (I^2^ = 0%).

**Table 3:** Health care surveys that compared identifiable and non-identifiable surveys

**Author, year**	**Population**	**Survey topic**	**Intervention**	**Control**
King, 1970	College students	Drug use	Coded questionnaire	Non-coded questionnaire
King, 1970	College students	Drug use and attitudes		
Campbell, 1990	General population	AIDS knowledge	Numbering with warning of reminders	Non-numbered questionnaire
Akl, 2011	Residency program directors (Family medicine)	Characteristics of residency program	Questionnaire numbered “to avoid sending reminders”	Non-numbered questionnaire
Akl, 2011	Residency program directors (Internal medicine)			
Kundig, 2011	Hospital staff	Patient safety culture	Numbered questionnaire	Non-numbered questionnaire
This study	Hospital doctors and nurses	Regret associated with health care	Numbered envelope	Non-numbered envelope

## Discussion

We found that numbering return envelopes in a survey about health care provider regrets has significantly increased the proportion of explicit refusals to participate and reduced the overall survey response rate. The reduction in the proportion of participants of 4.7% did not reach the pre-specified level of statistical significance of 0.05 (p = 0.073). This constitutes weak evidence against the hypothesis of no effect. Pooling this result with similar observations made in previous studies suggests that numbering questionnaires reduces participation by approximately two to three percentage points.

Whether this decrease is important enough to affect survey data collection procedures is debatable. Numbering questionnaires or return envelopes allows the researcher to send reminders only to those who have not yet responded. This reduces costs, and avoids the irritation some respondents may feel when they receive an unnecessary reminder. A small reduction in the response rate may be an acceptable price to pay for these benefits. On the other hand, response rates in mail surveys are typically lower than desirable, and may be declining [16,17]. Any measure that increases the response rate of a survey and its representativeness of the target population is welcome. Optimal response rates can be obtained only through an accumulation of design features that yield marginal gains, including a clear explanation of the utility of the survey, an attractive layout, incentives, and reminders [2]. It appears that the assurance of confidentiality may be another useful measure.

It is unclear whether numbering survey returns plays a role in all surveys or only in those instances where confidentiality is particularly important. We tested the effect of numbering in a sensitive survey. Regrets felt by doctors and nurses may be related to errors or to communication issues when providing healthcare. A potential respondent may well decide that it may not be prudent to participate and run the risk of being identified. Among previous studies of the effect of numbering, one concerned medical residency programs, which is not a particularly sensitive topic, and others pertained to drug use, AIDS knowledge, and patient safety climate. The meta-analysis did not show any amount of heterogeneity. There is thus far no evidence that the degree of sensitivity of the survey topic modifies the effect of questionnaire numbering.

Regardless of the effect of numbering, this survey was notable by its low response rate of 31.7%. While such response rates are not uncommon (e.g., [18]), participation was considerably lower than in several surveys of doctors and nurses conducted at the same hospital in recent years, which typically yielded response rates between 50% and 60% [19-21]. We believe that the topic of the survey – regret related to health care – was the chief cause of this reluctance to participate. By definition a regretted situation is unpleasant to recall, and revisiting it in detail and sharing these details with others may be too much to ask for, whether confidentiality is guaranteed or not. As evidence of an unusual and possibly emotional response pattern, we have not previously seen explicit refusals approaching the proportion of 21.4% observed in this study; in our experience less than one tenth of the eligible sample will check the “I do not wish to participate” box in a typical survey – e.g., about 5% in a survey of health insurance plan members [22]. Limited evidence suggests that people who explicitly refuse to participate in a survey may differ from those who fail to respond [23]. Our results suggest that regret related to providing health-care may have been a too sensitive topic for many potential respondents.

A low response rate may cause selection bias, but the numbering of return envelopes may also produce information bias, by causing the respondents to answer differently than they would have done in the absence of numbering. These 2 types of bias cannot be distinguished in our study. In comparisons of item responses, 12 of 105 differences between the numbered and non-numbered groups were statistically significant. However, the various survey items were mutually correlated, and a permutation test showed that this proportion (11.4%) did not significantly differ from the type 1 error rate of 5% that characterizes the null hypothesis of no bias. It is tempting to focus on the elevated proportion of significant differences observed for the regret intensity items (7 out of 19) and to conclude that response bias concerns these particularly sensitive items. However, any post-hoc selection of a subset of items increases the likelihood of misinterpreting type 1 errors as true differences. Of note, a low response rate does not always imply a large amount of bias [23]; e.g., in a patient opinion survey, the mean problem scores obtained after the first mailing, when only about 30% of questionnaires hada been returned, differed by less than one tenth of a standard deviation from the final results (difference 1.9 points, SD 23.8), when a response rate of 70% had been achieved [24].

This study has several limitations. First, the study was set up in the middle of an ongoing survey, prompted by an unusual response pattern, and not planned in advance. Therefore randomization was non-concurrent (numbered envelopes were sent first and non-numbered were sent two months later, even though both samples were drawn from the same randomly ordered lists). The delay could alter the outcomes if it changed exposure to external events that might influence survey response, such as a summer holiday period; however, this was not the case to our knowledge. Second, the sample size was driven by the primary purpose of the survey (data collection on regret), and not by a power analysis related to the comparison of numbered and non-numbered envelopes; as a result the size of the second (non-numbered) group was comparatively low. A post-hoc power estimation suggests that the power to detect a difference between 30% and 35% with the actual sample size was below 50%. Perhaps most importantly, we were not able to establish with certainty the reasons for the negative effect of envelope numbering, as we did not discuss the motivations of the respondents and non-respondents. The meta-analysis included only a small number of eligible studies, and had limited power to examine differences between survey identifiability methods (e.g., different effect of questionnaire numbering versus envelope numbering).

As our study employed paper questionnaires and postal delivery, the results are not directly applicable to electronic surveys. However, concerns about anonymity and traceability may be particularly important for electronic surveys, because online privacy management remains opaque and potentially untrustworthy for many users. This may contribute to the low participation rates regularly encountered in internet surveys e.g., [25].

## Conclusions

Our results, along with the meta-analysis of similar studies, indicate that the identifiability of completed questionnaires will lower the response rate of postal surveys by two to three percentage points. However, there was no strong evidence that identifiability causes bias. Further research should ascertain whether this effect depends on the surveyed population, the contents of the questionnaire, or other characteristics of the data-collection process.

## Competing interests

The authors declare that they have no competing interests.

## Authors’ contributions

TP planned the study, analyzed the data, conducted the systematic review, wrote the manuscript. SC and SR planned the study, managed the data collection, interpreted the results, revised the manuscript. TA, RS planned the study, interpreted the results, revised the manuscript. DC planned the study, conducted the systematic review, interpreted the results, revised the manuscript. CC conducted the meta-analysis, interpreted the results, revised the manuscript. All authors approve the manuscript.

## Pre-publication history

The pre-publication history for this paper can be accessed here:

http://www.biomedcentral.com/1471-2288/14/6/prepub

## Supplementary Material

Additional file 1 PRISMA checklist. Click here for file
